# Consequences of COVID-19 Confinement on Anxiety, Sleep and Executive Functions of Children and Adolescents in Spain

**DOI:** 10.3389/fpsyg.2021.565516

**Published:** 2021-02-16

**Authors:** Rocío Lavigne-Cerván, Borja Costa-López, Rocío Juárez-Ruiz de Mier, Marta Real-Fernández, Marta Sánchez-Muñoz de León, Ignasi Navarro-Soria

**Affiliations:** ^1^Department of Developmental and Educational Psychology, University of Malaga, Málaga, Spain; ^2^Department of Health Psychology, University of Alicante, Alicante, Spain; ^3^Department of Developmental and Educational Psychology, University of Alicante, Alicante, Spain

**Keywords:** executive functions, sleep, anxiety, adolescents, children, confinement, COVID-19

## Abstract

Children and adolescents are not indifferent to the dramatic impact of the COVID-19 pandemic, and the need to be forced to live in confinement. The change in life to which they have been abruptly subjected forces us to understand the state of their mental health in order to adequately address both their present and future needs. The present study was carried out with the intention of studying the consequences of confinement on anxiety, sleep routines and executive functioning of 1,028 children and adolescents, aged from 6 to 18 years, residing in Spain to; assess if there are differences regarding these consequences in terms of sex and age; how anxiety affects executive functioning in males and females; and to examine the possible correlations between the measured variables. For this purpose, an online questionnaire containing five sections was designed: the first section gathers information on sociodemographic and health data, while the following sections gather information from different standardized scales which measure anxiety, sleep and executive functions, whose items were adapted in order to be completed by parents, and/or legal guardians. The statistical analyzes carried out highlights significant differences in executive functioning between males and females. In turn, in regards to age, greater difficulties were detected in anxiety in the 9 to 12 age group and greater sleep disturbances between 13 and 18 year olds. On the other hand, significant differences were found in intra-sexual executive functioning depending on whether they presented greater or lesser anxiety, with executive functioning being more tendentiously maladjusted in males than in females, revealing a significantly relevant effect size (*p* = 0.001; *ω^2^* = 0.27 BRIEF-2; *ω^2^* = 0.19 BDEFS-CA; 95%). Positive correlations are obtained between state anxiety and sleep and executive functioning alterations. Finally, through Path Analysis, it is verified that state anxiety is the variable with the greatest weight within the model that would explain the alteration in the executive functioning of the present sample.

## Introduction

On March 11, 2020, the World Health Organization (WHO) stated a global pandemic caused by COVID-19. Since the beginning of the alert until 24 of May 2020, 235.772 cases of COVID-19 have been reported in Spain, 1.976.120 (Red Nacional de Vigilancia Epidemiológica. Conserjería de Salud and de Epidemiología, 2020) in Europe and 5.165.481 worldwide. Given the rapid spread of the virus, all countries have had to take measures to prevent the collapse of their respective health systems. The WHO suggests social isolation, limitation of mobility and quarantining the population as the most effective strategy in containing and mitigating the speed of transmission of the infection. From March 14 to June 21, 2020 a state of alarm was declared in Spain and with it the closure of many workplaces, including primary schools, secondary schools and universities (Royal Decree 463/2020, of March 14, BOE N°. 67; España, 2020). Since then, the entire population has been forced to remain homebound for long periods of time. Many adults have experienced alterations in their work, financial, and personal situation. In addition, children and adolescents have completely restricted social contact with their peers and significantly limited their physical activity (Danese et al., 2020), causing major changes in their daily routines, and what was previously considered a normal day is now unfeasible. In record time, the population has been forced to adapt to new living conditions unthinkable a week before the onset of state of alarm.

The growing number of patients, the expansion through different provinces and the unpredictable future of the epidemic have raised the concern of the entire population. In addition, other collateral damage of an economic, employment, social and personal nature, such as the loss of a job or loved ones has aggravated the situation (Danese et al., 2020). All of this has caused high psychological pressure on people in different countries (Duan and Zhu, 2020), with the mental health of adults, children and adolescents being diminished, producing changes in mood, behavior and other daily habits (Chen et al., 2020; Li S. W. et al., 2020; Yang et al., 2020).

A sector of society that is highly susceptible to vulnerability is the child and adolescent population since the contexts in which they live and develop are altered. Although there are few studies on the responses of this population to epidemics (Klein et al., 2009), there is a large body of research that shows that children and adolescents in situations of prolonged stress may experience immediate or long-term mental health problems (Laor et al., 1997; Hoven et al., 2005; Plourde et al., 2017; Han and Lee, 2018; Bender et al., 2020; Park et al., 2020). Quarantine and hospitalization of the child or their family members are conditions that can generate high levels of stress, in the same way limited access to mental health services during quarantine can aggravate previous psychological problems or lead to new difficulties (Espada et al., 2020). Emotional stress has neurobiological consequences that increase the probability of exacerbating concomitant illnesses and the vulnerability to meeting criteria for a mental disorder such as anxiety disorders, depression, sleep disorders, and acute stress, among others (Asmundson and Taylor, 2020; Bender et al., 2020; Ji et al., 2020; Vahia et al., 2020).

Mood disorders, in their different types and varieties, constitute one of the most diagnosed psychological problems in children and adolescents in Spain, as well as being the highest demand for care within Infant-Youth Mental Health Units, with rates between 20 and 39% for conduct disorders; between 17 and 26% for anxiety disorders; between 4 and 14% for depression; and around 12% for developmental disorders (Bernstein et al., 1996; Aláez et al., 2000; Echeburúa and de Corral, 2009). Their prevalence can be considerably affected depending on different variables (age, previous and/or parental psychopathology, psychosocial stressors, etc.), which can condition the severity or duration of the symptoms and predict the evolution toward more severe pathologies in future developmental stages (Bragado and Bersabé, 2000; Orgilés et al., 2012), becoming a risk factor for depression (Huberty, 2012). Specifically, the extraordinary situation to which minors are exposed due to the long period of confinement in the home as well as the threats posed by the pandemic, together with their own personal and family circumstances, lead to a higher probability of the appearance and greater impact of symptoms related to this disorder in its different manifestations (Chen et al., 2020; Li S. W. et al., 2020; Yang et al., 2020).

Anxiety and depressive disorders have a complex and bidirectional relationship with sleep disorders (Johnson et al., 2000). Sleep is an evolutionary and active process that begins prenatally as a result of dynamic biopsychosocial balance; and its evolution depends on the harmony between these three factors (Pin-Arboledas and Lluch Rosello, 2011). The unprecedented situation that we are experiencing caused by COVID-19 affects said biopsychosocial balance. In the consensus document on the management of insomnia in childhood and adolescence prepared by Pin et al. (2017), it is concluded that 27% of children aged 5–12 years present resistance in going to sleep, 11% prolonged sleep latency, 6% frequent awakenings and 17% difficulties in getting up in the morning (Pin Arboledas et al., 2011). In adolescents, 38.5% present poor subjective sleep quality and 23.1% have sleep latency greater than 30 min (García-Jiménez et al., 2004). In addition, it is indicated that sleep-related problems can have a significant impact on both emotional and cognitive levels, as well as on learning.

Along with emotional and sleep difficulties, it is not unreasonable to think that cognitive processes can be affected in situations of confinement. It has been observed that stress, both chronic and acute, affects cognitive processes governed by the Prefrontal Cortex (PFC) (Park and Moghaddam, 2017). This area dominates the processes and functions of the executive system, including working memory, self-regulation of emotions, cognitive flexibility, organization and planning, decision-making, and goal-orientated behaviors among others (Lavigne, 2009; Lavigne and Romero, 2010; Sánchez et al., 2019). Several authors highlight the role of these functions, and especially those related to planning, as possible mediators between perceived stress and subjective memory complaints (Ruiz-Sánchez de León et al., 2010; Molina-Rodríguez et al., 2016). Molina-Rodríguez et al. (2018) further reveal that in stressful situations, perceived stress will be inversely related to the level of executive control and attention problems.

In typical adults, research suggests that anxiety interferes with efficient cognitive processing on tasks involving executive function (Eysenck et al., 2007; Shields et al., 2016). Also, acute stress impairs executive function in part by increasing noradrenaline (Alexander et al., 2007) and state anxiety is correlated with noradrenergic receptor occupation (Yu et al., 2008). Thus, anxiety may impair executive function by both draining cognitive resources and concurrently enhancing noradrenergic activity. Furthermore, PFC has a high level of neuroplasticity throughout life, being especially sensitive during childhood and adolescence (Munakata et al., 2004). Specifically, it has been observed that in these stages, behavioral stress can generate changes at both a structural and functional level in PFC by modulating neuronal, molecular and chemical connections (McEwen and Morrison, 2013; Bender et al., 2020).

Confinement leads to a loss of routine, a reduction in social and physical contact, frustration, boredom and a sense of loneliness that can be difficult to manage for many people (Rodríguez, 2020). Studies related to the effect of quarantine periods in other health crises such as the epidemic outbreak of Severe Acute Respiratory Syndrome (SARS) (2003), Ebola (2014) or influenza AH1N1 (2009, 2010), allow us to predict the psychological consequences that could be derived from the current crisis (Brooks et al., 2020). These studies showed a prevalence of anxiety symptoms of up to 20% and depressive symptoms of up to 18% in the quarantined population, with health workers being significantly more affected. Sprang and Silman (2013) found that children who have been quarantined during pandemic diseases are more likely to develop acute stress disorders, adjustment disorders, and pain. Thirty percent of the children studied in isolation or quarantine met the clinical criteria for post-traumatic stress disorder. A preliminary study conducted on a sample of 320 children and adolescents aged 3–18 years in Shaanxi Province during the COVID-19 epidemic by the China-EPA-UNEPSA collaborative working group (Jiao et al., 2020) showed that children aged 3 to 6 years old were more likely than older children to manifest symptoms such as ambivalent anxiety attachment and fear that family members could become infected. Children aged 6 to 18 were more likely to show inattention and persistent inquiry. Clinging, inattention, and irritability were the most severe psychological conditions demonstrated by children in all age groups. Rates of fear, anxiety, and other emotions were higher in children residing in highly epidemic areas; however, the differences between areas identified by different levels of epidemic risk were not statistically significant. All these studies conclude that significant behavioral changes such as maintaining hyper-alertness, excessive handwashing and crowd avoidance are detected after quarantine period. Therefore, the data points to the fact that there are groups that have suffered and will suffer more from confinement such as; those with previous mental pathology, pregnant females, the elderly, health workers, and children and adolescents (Daley, 2020).

The National Child Traumatic Stress Network (2020) states the psychological response to COVID-19 will change with age. In the preschool stage, manifestations of fear, loss of appetite, increased tantrums and complaints or ambivalent anxious attachment behaviors (among others) are expected. At ages 6 to 12 years, higher rates of irritability, nightmares, sleep and appetite problems, somatic symptoms, or loss of interest in peers, as well as excessive attachment to parents, may occur. In adolescents from 13 to 18, in addition to physical symptoms, sleep problems or isolation, increased or decreased energy, higher rates of apathy or inattention to behaviors related to health care are expected.

Given the seriousness of the situation surrounding the mental health of children and adolescents, we intend to carry out this study with the following objectives: (1) To understand the consequences of confinement by COVID-19 on anxiety, sleep and executive functioning (planning/organization, self-regulation of emotions, flexibility, time management, organization/problem solving, inhibition/containment and motivation) of children and adolescents; (2) To check whether there are differences in these consequences in terms of sex on the one hand and age on the other; (3) To assess how anxiety affects executive functioning in males and females; and (4) To examine possible correlations between the variables that have been measured on the mental health of children and adolescents.

## Materials and Methods

### Design

The design is multimodal, since it will include different types of methodologies according to the objectives of this study. Thus, an associative-comparative and explanatory design was used to carry out this research (Ato et al., 2013).

### Participants

The initial simple was compromised of 1,033 children and adolescents, of which 1,028 (548 males, 478 females and 2 people who did not identify as either) finally participated, with ages between 6 and 18 years (*M* = 10.34; *DT* = 3.64). The sample was recruited after sending telematic questionnaires and the inclusion criteria consisted of being in quarantine due to the COVID-19 pandemic, residing in Spain and being between the ages of 6 and 18. The collaboration of the legal guardians (*N* = 1028) was required, with them answering the questionnaires in relation to what was observed during confinement or asking their children when necessary. Of these, 868 were females and 160 males, aged between 19 and 68 years (*M* = 42.94; DT = 6.78). All the legal guardians were aware of the different phases and characteristics of the study, signed the informed consent and completed the questionnaire. In the case of people over 18, they could sign the informed consent and complete the questionnaire themselves. Those who did not fully complete the questionnaire or did not provide the informed consent were excluded from the study.

The nationality of the respondents was mostly Spanish (95.9%). The relationship that these people have with the child or adolescent in 84.6% is maternal, in 12.8% paternal, in 1.4% of siblings and the rest with percentages ranging between 0.1 and 0.6%, other relationships such as grandparents, uncles, neighbors’ and/or guardians.

### Instruments

The instrument, designed to be completed by parents and/or legal guardians of children and adolescents from 6 to 18 years of age, takes approximately 15–20 min to complete and consists of four sections that include: (1) socio-demographic and health-related data, (2) state/trait anxiety in children and adolescents, (3) sleep, and (4) executive functioning. The first section has been elaborated *ad hoc* based on demographic and health variables that are mainly related to the contextual characteristics of minors, given the current situation. The subsequent sections gather information from different standardized scales whose items have been used and adapted in order to be completed by adult observers.

#### *Ad hoc* Questionnaire of Socio-Demographic and Clinical Data

Designed to collect information regarding sociodemographic variables such as level of education attained by informants, the monthly income of the family, the employment situation of the informant and their partner before and after the declaration of the state of alarm, employment sector to which they belong, size of the house in which they live during quarantine and exterior spaces which the house possess (window, balcony, terrace, patio, garden or exterior terrain). In addition, information was collected regarding the child’s mental health, as well as whether he or she was taking medication. Lastly, it was asked whether the family unit included persons with risk factors for contracting coronavirus and whether any members had tested positive for the disease.

#### State-Trait Anxiety Inventory for Children, STAIC (Spielberger, 1973)

This questionnaire is derived from the State-Trait Anxiety Inventory (STAI) test. The Spanish version is applied, adapted for the first time by Spielberger et al. (1982) and currently revised on a psychometric level by Guillén-Riquelme and Buela-Casal (2011). Thus, there is a solid evidence in relation to the measured constructs, because of its long history of use in different fields (Barnes et al., 2002; Rossi and Pourtois, 2012; Guillén-Riquelme and Buela-Casal, 2014) and updated to the general Spanish population (Spielberger et al., 2015). It evaluates anxiety as a state and as a trait, constituting two independent scales made up of 20 items each with 3 response options (1 = not at all, 2 = somewhat, 3 = very much). The first scale refers to how the child feels at a given time, and the second relates to how he/she feels in general. The state anxiety scale tries to clarify “how the child feels at a given moment,” and measure transitory states of anxiety, that is, feelings of apprehension and tension and concern which fluctuate in intensity over time. Examples of items used in this scale are “your child feels calm” or “your child feels restless.” The trait anxiety scale measures “how the child feels in general,” exploring relatively stable differences in propensity to anxiety, that is, differences between children in their tendency to show anxious states. Some items on this scale are “your child is concerned about making mistakes” or “your child feels like crying.” Although the original implementation of the instrument is self-applicable with help of tutors or guardians if necessary, due to the special circumstances of the confinement and the telematic sending of the test, it was chosen that the guardians would be the ones to complete the questionnaire, in collaboration with the child when necessary. For this, the formulation of each item was adapted as previously exemplified. However, in order to guarantee the reliability of this instrument, Cronbach’s alpha was calculated for each subscale. Thus, for State Anxiety, the internal coefficient is 0.91 and 0.89 for Trait Anxiety.

#### BEARS. Screening for Sleep Disorders in Childhood (Owens and Dalzell, 2005)

A brief sleep screening test made up by 9 items which analyses five main areas: bedtime problems (B-), excessive daytime sleepiness (E-) awakenings during the night (A-), regularity and duration of sleep (R-) and snoring (S-). The aim of this test is to detect sleep disorders in children and adolescents between the ages of 2 and 18. This test was completed by parents/guardians answering questions such as “does your child have trouble going to bed?” or “does your child have trouble waking up in the morning, does he/she seem sleepy during the day or take naps?” Each item had 7 response options, with 1 = totally disagree and 7 = totally agree, even though originally a dichotomous response was used. It was decided in the following way, since it is more appropriate at methodological level and thus provides greater relativity in the type of response. Regarding the reliability and internal consistency, the scale presents a Cronbach’s alpha coefficient of alpha = 0.732 (Ramírez-Vélez et al., 2018).

#### Behavioral Evaluation of Executive Function: BRIEF-2 (Gioia et al., 2017). Spanish Adaptation

This tool collects information from children between the ages of 5 and 18, and assesses executive behavior in the natural environment by providing an ecological perspective of such functions. It consists of nine scales made up of 63 items with three possible response options (always, sometimes or never). These 9 scales provide measures regarding inhibition, self-monitoring, flexibility, emotional control, initiative, working memory, planning, and organization, task supervision and organization of materials (Gioia et al., 2017). These domains are adjusted forming in turn three indices: behavioral regulation, emotional regulation and cognitive regulation. In addition, it provides a global index of executive function. It has two versions that share the same structure, BRIEF 2-Family, which is answered by family members (mother, father or others) and BRIEF-2 Teachers, to be completed by the teaching staff. Given the length of the original questionnaire and the situation due to confinement, it was decided for the present work to carry out a brief screening selecting 12 items from the family version that were directly related to the objectives of the study. For this, 6 of the 8 items of the control subscale were used, 4 of the 8 items of the flexibility subscale, and 2 of the 8 items of the planning and organization subscale. Due to the selection of items for this study, the discrimination indexes of these items are presented as indicators of the reliability of the test for each of the subscales; specifically, the items that compose the subscale of emotional control have a well-defined discrimination index that ranges from 0.46 to 0.72; the items selected from the subscale of flexibility also have a good discrimination quality between 0.29 and 0.37; and something similar exists in the items selected from the subscale of planning and organization, where there is an excellent discrimination of 0.56 in both items.

#### Barkley Deficits in Executive Functioning Scale. Children and Adolescents (BDEFS-CA), Short Form (Barkley, 2012)

l This scale is completed by the child’s parents/guardians and evaluates information about deficits in the executive functioning of children and adolescents (between 6 and 17 years old), in daily life activities. It is composed of 20 items with four response options assess the frequency in which behavioral alterations occur (never, sometimes, often, and very often). It consists of items such as “your child wastes or mismanages his/her time” or “your child has trouble planning or preparing for upcoming events.” Regarding the reliability and internal consistency, the short version of the scale presents a Cronbach’s alpha coefficient of alpha = 0.732. This version constitutes a screening of the extended questionnaire (70 items), both of which bring together information from different executive domains (time management, organization/problem solving, inhibition/containment, motivation, emotional regulation).

### Procedure

A single evaluation was carried out by means of an online questionnaire that combined all of the instruments. This format was chosen because of the confinement situation decreed in the country. The sample was selected based on age and COVID-19 alarm status.

On Thursday, April 16, 2020 at 7:30 p.m., the questionnaire was sent out and disseminated through social networks and local media requesting citizen collaboration to participate in the study. The questionnaire has been online for 2 weeks, closing on April 10 at 00:00 p.m.

At the beginning of the instrument, the informed consent of the parents and/or legal guardians of the participants was required, who were informed of the anonymity of their data and the exclusive use for which they were to be used. Written informed consent was obtained from the parents of all participants. In addition, the ethical principles of the Helsinki declaration were respected (World Medical Association, 2013). This study was carried out in accordance with the Ethics Committee and Vice-rectorship for Research and Knowledge Transfer of the University of Alicante in Spain (UA-2020-05-12).

### Statistical Analysis

To carry out the statistical analysis, we started with the refinement of the database. For this, an exploratory analysis was carried out, duplicated cases were eliminated and inverse items were recoded.

A quantitative methodology was carried out in which frequency and descriptive analyses (mean, median, and standard deviation) were applied for the sociodemographic variables (age and sex of the participants) and the variables state anxiety, trait anxiety, sleep disturbance and executive functions. In this way, a statistical criterion was used to group the different variables into low, average and high scores, by taking the 33.3 and 66.7 percentiles as cut-off points to find out the frequency of each group, in addition to making an expert judgement by professionals from the University of Malaga and the University of Alicante. Likewise, direct scores were used to calculate a total sum (including all the items of each variable) for each of the subjects as well as an arithmetic mean to measure the average alteration of the total sample in each variable. Furthermore, prior to the analysis of differences and in order to choose the most suitable hypothesis test, the assumptions of linearity (scatter diagrams), normality (Kolmogorov-Smirnov test), independence (runs test) and homoscedasticity (Levene test) were checked (Field, 2009).

In addition, difference analyses were performed by means comparison. The parametric Student’s *t* test was applied to two independent samples in order to check the differences by sex in the variables state anxiety, sleep and executive functions. ANOVA was performed to find the differences between the age groups (the sample was segmented into 3 age groups: 6–8 years, 9–12 years, and 13–18 years). Subsequently, Tukey’s *post hoc* test was applied to check between which groups there were statistically significant differences. The trait anxiety variable did not meet the statistical assumptions and, therefore, the non-parametric Mann-Whitney *U* test was applied for differences by sex and the Kruskal-Wallis *H* test for differences by age group. Likewise, ANOVA was applied to check if there were statistically significant differences in sex with low, medium and high anxiety, as well as intra-sex between males with different levels of anxiety and in the same way for females. The effect size was obtained through the calculation of Cohen’s d to determine the magnitude of differences by sex with respect to executive functions. Hays’s omega for differences by age groups in state anxiety and sleep, and the differences between anxiety groups in executive functions. The interpretation of the effect size was according to Cohen (1988). A confidence level of 95% was established (Field, 2009).

Finally, the correlations between the children’s sex, age, state anxiety, trait anxiety, sleep and executive functions were calculated, according to the interpretation of Hernández-Lalinde et al. (2018), and a Path Analysis was carried out to create a possible model to explain the influence of state/trait anxiety and sleep variables in executive functioning (measured with BRIEF-2 and BDEFS-CA).

All data were analyzed by a member of the team with experience and training in methodology, using SPSS Statistical software package, IBM SPSS Statistics v25.

## Results

The results obtained after the telematic completion of the above-mentioned questionnaires by the legal guardians of 1,028 children and adolescents aged 6 to 18 years (*M* = 10.34; SD = 3.64), who were living under a situation of confinement in Spain due to the COVID-19 pandemic, were analyzed. Firstly, the average scores on the different scales used for the research have been calculated and compared with the average scores which appear in the test manuals, understanding the latter group as a normative non-confined population. The differences between the scores obtained by the unconfined sample and the confined sample are visibly different (Supplementary Table 1).

With this in mind, can be observed in Supplementary Figure 1, the percentage of total sample (*N* = 1028) with low, medium and high scores in each of the administered tests.

In order to test for differences in the mental health consequences of the COVID-19 confinement for children and adolescents based on sex, a *T*-test for two independent samples and the non-parametric Mann-Whitney *U* test were performed, as can be seen in Supplementary Tables 2, 3.

In addition, with the aim of checking whether there are differences in the consequences of confinement by COVID-19 on the mental health of children and adolescents depending on their age, a one-way ANOVA and the non-parametric Kruskal-Wallis *H* test were applied. Supplementary Tables 4, 5 show the distribution of the sample into three homogeneous groups depending on the developmental stage in which they can be found.

On the other hand, differences between males and females with low, average and high state anxiety (SA) in relation to executive functions can be seen in Supplementary Tables 6, 7.

With the aim of studying the relationships between the state/trait anxiety variables and the rest of the variables measured on the mental health of children and adolescents, a correlation study was carried out (Supplementary Table 8). The interpretation is made according to the range of values established by Hernández-Lalinde et al. (2018).

Finally, with the purpose of finding out how variables such as State/Trait Anxiety and Sleep quality influence the executive functioning of children and adolescents a Path Analysis was performed (see Supplementary Figure 2).

## Discussion

In recent months, the study of the impact of COVID-19 pandemic on different variables has, as can be expected, aroused great interest in scientific community. However, most of these studies either use the entire population as their target sample, or remove children and adolescents, even though these are the people who will constitute the future of our society and are a group of high vulnerability. Children and adolescents are not indifferent to the dramatic impact of the COVID-19 epidemic. The change in life to which they have been abruptly subjected forces us to understand the state of their mental health, in order to adequately address both their present and future needs.

Firstly, we would like to highlight the differences found between the means obtained by the subjects of the sample of the present study, who are in a situation of confinement at the time of proving their answers, and the results obtained by other samples that are not confined. The clearest differences are observed in State-Anxiety (Unconfined = 31.2 and Confined = 34.7), in the quality of sleep (Unconfined BEARS = 7.4 and Confined BEARS = 13.18) and in difficulties in Executive Functions measured with BDEFS-CA (Unconfined = 33.2 and Confined 70.69). Based on these data, it is unquestionable that the confined population that forms part of the studied sample shows, in comparison with non-confined samples, greater deterioration in anxiety, rest and effectiveness in executive functions. This fact justifies the statements that will be carried out as a conclusion of the present investigation.

The results of the present study show that out of a sample of 1,028 children and adolescents, 66.9% and 67.9% present medium to high scores in trait and state *anxiety*, respectively. In relation to *sleep*, 40% show medium scores, 36.4% high and only 23.6% low scores. In regards to *executive functioning*, we found that between 67.1 and 68.3% of children and adolescents show medium to high scores. These findings are in line with those found by Jiao et al. (2020) who, using a sample of 320 children and adolescents between the ages of 3 and 18 and with the aim of detecting behavioral and emotional disorders during confinement, found that the most common problems presented were those related to attention, irritability, fear of asking about the coronavirus, and ambivalent attachment behaviors. However, sleep problems, lack of appetite and hyperactivity were also detected. With these findings, the researchers aim to detect people who are susceptible to pathology in order to diagnose and subsequently treat them. On the other hand, studies have also been conducted with adult population during the confinement due to the COVID-19 pandemic, in which the presence of mental health problems has also been detected. A study carried out in China by Cao et al. (2020) with university students during the beginning of the pandemic found that 24.9% had severe-moderate to medium anxiety (0.9, 2.7, and 21.3%), as well as verifying that social support negatively correlates with the anxiety levels (*p* < 0.001), suggesting monitoring this population during epidemics, as to avoid deterioration in mental health. Likewise, Li S. W. et al. (2020), in a study carried out with 17,865 adult participants, found that negative emotions such as anxiety, depression, outrage, and sensitivity to social risks increased, while positive emotions and feelings such as happiness and satisfaction with life decreased. The conditions that social isolation generates are configured as a non-normative stressor that increases the possibility of presenting mental problems for the first time or the exacerbation or recurrence of pre-existing mental disorders (Asmundson and Taylor, 2020).

In order to study differences health of our sample depending on sex, as shown by the analyzed results, being male or female does not determine the presence in the studied sample of more or less anxiety or sleep problems. However, statistically significant differences are found in executive functioning, in favor of women, with a small effect size, so they would not be considered clinically relevant. In other words, male children and adolescents show more alterations in planning and organizing, ability to self-regulate their emotions, manage time well, solve problems, be motivated, adapt to different circumstances, and inhibit inappropriate behavior.

Research conducted with the intention of determining the existence of differences in executive functioning regarding sex, shows non-congruent findings. There are studies with population of up to 6 years old that find better levels of executive functioning in females than in males, especially in inhibitory and emotional control and resistance to delay (Matthews et al., 2009). In contrast, other studies have failed to find such differences (Ferreira et al., 2015). In a study carried out in Spain with a sample of 66 students aged between 5 and 6 years old, using the BRIEF-P scale, Romero-López et al. (2016), it was found that females obtained significantly higher scores than males in inhibition, memory and planning, however, males obtained higher scores in flexibility. No significant differences were found in the remaining variables that were analyzed on executive functioning. Studies with adolescents indicate the same controversies; some find a difference between adolescent males and females (Sant’Anna et al., 2007) and some do not (Archana et al., 2014). However, these studies were not carried out in situations of confinement. One study that was carried out under these circumstances, but with a sample of 1,210 adults instead of children, was the study conducted in China by Wang et al. (2020), which found that being a woman, among other variables such as being a student, having previous symptoms such as myalgia, dizziness, coryza and a poor perception of one’s health, is associated with a greater psychological impact and high levels of stress and symptoms of anxiety and depression. Likewise, in another study carried out with adult Chinese population, it was also found that women presented a higher level of anxiety than men (Lozano-Vargas, 2020).

Regarding the variable of age, the data show differences between the groups. On the one hand, the pre-adolescents and adolescents obtained higher levels of anxiety, while for the variable of sleep adolescents were those who showed more alarming scores, with small effect sizes as indicated. The findings related to anxiety are in line with other authors who indicate that from pre-adolescence onwards, anxiety problems are accentuated, specifically generalized anxiety disorders (García-López et al., 2001; Kendall and Pimentel, 2003; Orgilés et al., 2012). In contrast, in younger children there are symptoms related to separation anxiety (Orgilés et al., 2011). Given that the confinement situation involves being at home with their parents, it is not uncommon for younger children to feel more protected, and therefore less anxious. In addition, adolescents’ perception and understanding of the problem is more direct and real than in younger children who are usually guided by their parents.

On the other hand, the results indicate that adolescents, unlike children and pre-adolescents, show significant sleep disturbances. Various studies postulate that age and hours of sleep are naturally inversely related (Gibson et al., 2006; Velayos, 2009). Furthermore, factors such as decreased physical activity, changes in routine and the precipitation of stressful events can cause sleep disturbances (Âkerstedt, 2006; Kredlow et al., 2015; Willis and Gregory, 2015). Some studies also point out that in adolescence other elements such as the use of electronic resources such as mobile phones, computers, and time on social network, contribute to the decrease in quality and hours of sleep (Billieux, 2012; Sahin et al., 2013). Specifically, in the study carried out by Rodríguez (2016) it was observed that the pattern of use in pre-adolescents is considerably lower than that of adolescents, thus causing alterations in the sleep-wake cycle, especially in the latter group. Furthermore, the absence of social contact with peers due to the confinement situation could lead to an increase in technology consumption, problems in falling asleep and difficulties in concentrating and achieving an adequate academic performance (Lin et al., 2011; Wang et al., 2011; Tresáncoras et al., 2017; Piqueras and Carrasco, 2018).

As the results regarding anxiety and executive functions show, we observed that the anxious symptoms associated with the period of confinement can justify a lower performance in the subjects’ executive functioning. Specifically, when comparing groups of males and females who present high levels of anxiety, a significant difference is observed in executive functioning (which are especially pronounced in men). Likewise, when analyzing groups with or without manifestations of anxiety, significantly lower scores in executive functions were observed in those groups (both males and females) that present higher levels of anxious symptoms, compared to those without anxiety. In all cases with a clinically significant effect size. As mentioned previously, the general population in countries such as China has suffered, in more than 50% of cases, the impact of the psychological symptoms of the pandemic. Specifically, 28.8% have experienced anxious symptoms (moderate-severe), throughout the first weeks of quarantine (Lozano-Vargas, 2020). However, we still do not have sufficient data regarding the emotional and cognitive consequences that this situation is having on younger age groups.

Along these lines, the work of Bragado and Bersabé (2000), carried out with three groups of children and adolescents: 67 subjects (with symptoms compatible with any type of anxiety disorder), 101 cases (clinical control group), as well as 155 (control group without pathology), aged between 6 and 17 years old, explores some of the risk factors associated with the detection of anxiety disorders. The results are especially relevant between the anxious group and the neurotypical controls, observing significant scores that point to stressful events, academic difficulties or psychopathological state of the parents as risk factors, among others. These types of factors allow discriminating between children with anxiety and children without pathology up to 85.58% using a multiple regression model.

In line with our results, the work of Márquez (2007) justifies the complexity of the neural substrate of anxiety and its relationship with cognitive mechanisms such as memory and executive functions, as well as adding that patients with generalized anxiety disorder manifest problems related to their poor executive capacity (notorious in deficits to plan, decision making or problem solving, among others). Moreover, executive disorders tend to have a higher prevalence in men during childhood and adolescence, being likely due to the strong association they show with various mental and neurodevelopmental disorders such as (Bausela-Herreras, 2012): attention deficit hyperactivity disorder, autism spectrum disorders, traumatic brain injuries, epilepsy, etc.

Likewise, the systematic review carried out by Langarita and Gracia (2019) addresses forty articles with a total sample of 1,098 cases that present symptoms compatible with generalized anxiety. Its aim is to explore the neuropsychological profile of subjects diagnosed with this disorder. Regarding executive functions, significantly lower scores are observed in cognitive flexibility (Tempesta et al., 2013), decision making (DeVido et al., 2009), regulation of emotions with negative valence (Kerns et al., 2014) and cognitive inhibition (Kalanthroff et al., 2017), however, no significant differences were found in behavioral inhibition between groups.

Finally, and with the aim of studying the correlations between the variables on the mental health of children and adolescents that have been measured, the data found supports the hypothesis that there is a strong correlation between state anxiety and sleep disturbances, on the one hand, and executive functioning on the other. However, although a correlation was found between all variables and trait anxiety, this correlation was not as strong. This would explain that state anxiety was the variable with highest weight (0.34 and 0.25) within the model that would explain the low scores in executive functioning of the present sample (see Supplementary Figure 2). These data suggest that the confinement situation due to the current pandemic may be affecting anxiety in the 6–18 year old population, which in turn may affect sleep and negatively impact executive functioning. However, the state of anxiety, sleep disturbances and alterations in executive functioning can vary over time and fluctuate in intensity, and even more so in this current situation. Psychological reactions to a pandemic are usually acute, and long-term emotional consequences can be observed (Li Z. et al., 2020). There is no doubt that due to the confinement caused by the current pandemic we are detecting changes in the mental health of children and adolescents from 6 to 18 years old. Coinciding with the reflections of Sprang and Silman (2013), it is recommended that all children and adolescents who present symptoms of anxiety, depression, sleep problems, executive disorders, among others, receive a psychological or psycho-pedagogical follow-up after the pandemic. However, if during the pandemic these symptoms are detected, something must be done to avoid this situation continuing after the post-pandemic, therefore, it would be recommended that those responsible for socio-educational and health policies -in this case linked to child and youth mental health-, implement public programmes that help prevent and/or treat the problems that the COVID-19 pandemic may generate on the mental health of children and young people.

Therefore, the results obtained in the present study contribute theoretically to knowing how and which people may be most affected during the confinement situation. This will allow in practice to detect early possible alterations in the mental health of children and adolescents, and initiate an early response in future situations of pseudo-confinement or confinement.

As the main limitation of the present study, we highlight the lack of a pretest or retest on the study sample, data with which it would be possible to compare to what degree the scores obtained are mainly generated by the current situation.

Derived from this study, three future investigations are projected that would complement the obtained results. First, to carry out a new collection of information for the same sample, once the current situation has been overcome, and the subjects of the study have recovered part of the psychosocial habits that characterize their day to day lives. Secondly, it would be very interesting to collect information about the cognitive ability of the subjects, in order to carry out a new study on the explanatory model, including this variable due to its determine nature when explaining the development of executive functions. Thirdly, to develop intervention programs to prevent and/or treat the difficulties found in the subjects surveyed with the aim of minimizing the possible consequences that confinement has generated, both in their levels of anxiety and quality of sleep, as well as in their executive functioning. The actions could be oriented toward the design and development of social and educational strategies that allow sufficient health measures to be carried out, without disrupting the life and routine of the most fragile population, children and adolescents.

Therefore, the perspectives of the present study are multiple. Firstly, it seeks to delve into the possible consequences in medium-long term that confinement may be causing in child and youth population. In addition, it is desired to expand this work to understand other variables that may be influencing the factors studied. And finally, it is intended to provide information to different government agencies in order to develop future protocols and action programs.

## Data Availability Statement

The datasets generated and analyzed for this study are available on Google Drive at: https://drive.google.com/drive/folders/1tDOMFKMnhsz6dLp0SEiT7jjpfN604PYV?usp=sharing.

## Ethics Statement

The studies involving human participants were reviewed and approved by the Ethics Committee of the University of Alicante. Written informed consent to participate in this study was provided by the participants’ legal guardian/next of kin. Written informed consent to participate in this study was provided by the participants’ legal guardian/next of kin.

## Author Contributions

RL-C, IN-S, RJ-RM, BC-L, MR-F, and MS-ML collected and processed the experimental data, performed the analysis, drafted the manuscript, and designed the figures. RL-C and IN-S were responsible for conceiving the idea of the study. IN-S and RL-C were involved in planning and supervising the study. BC-L and MR-F were responsible for most of the technical aspects of the study and conducted the statistical analyses. All authors contributed to the article and approved the submitted version.

## Conflict of Interest

The authors declare that the research was conducted in the absence of any commercial or financial relationships that could be construed as a potential conflict of interest.
