# Supplementary material for: Consequences of COVID-19 Confinement on Anxiety, Sleep and Executive Functions of Children and Adolescents in Spain
Source: Front Psychol. 2021 Feb 16;12:565516. doi: 10.3389/fpsyg.2021.565516 (PMC7921483; doi:10.3389/fpsyg.2021.565516)
Supplement: Supplementary file 1 [file Image_1.pdf]

Supplementary Material

1 Supplementary Figures and Tables

1.2. Supplementary Figures

Figure 1. Percentage of total sample (N=1028) with low, medium and high scores in each of the administered tests.

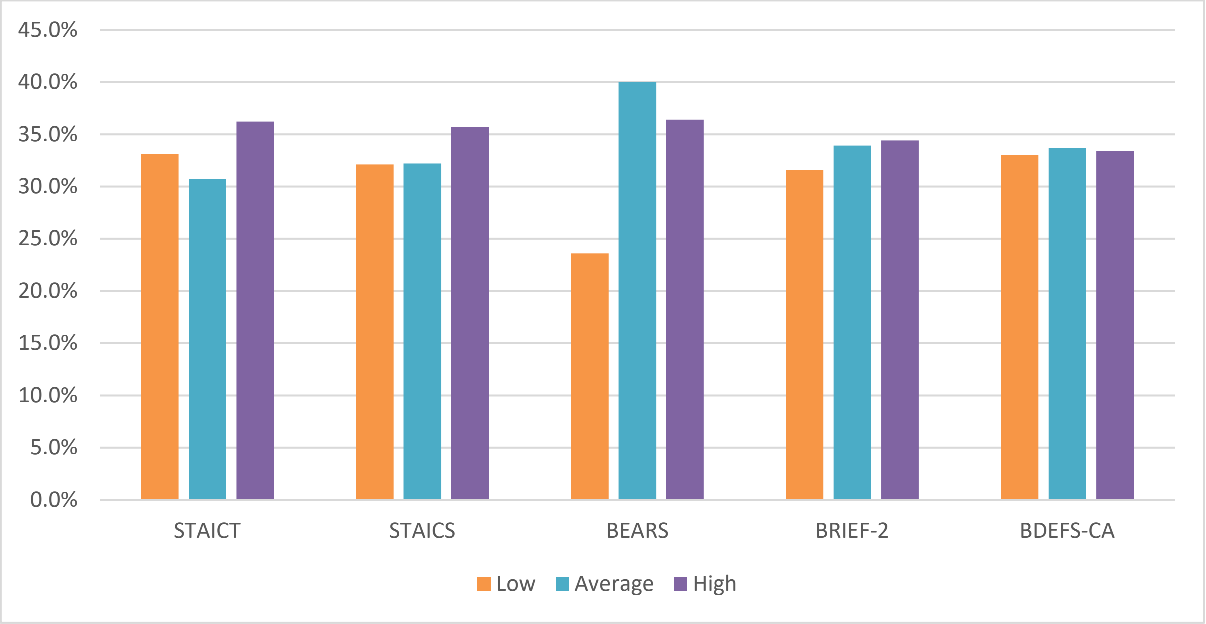

STAICS, State Anxiety Inventory for Children.

STAICT, Trait Anxiety Inventory for Children.

BEARS. Screening for sleep disorders in childhood.

BRIEF-2. Behavioral Evaluation of Executive Function.

BDEFS-CA. Barkley Deficits in Executive Functioning Scale. Children and Adolescents.
