# Supplementary material for: Consequences of COVID-19 Confinement on Anxiety, Sleep and Executive Functions of Children and Adolescents in Spain
Source: Front Psychol. 2021 Feb 16;12:565516. doi: 10.3389/fpsyg.2021.565516 (PMC7921483; doi:10.3389/fpsyg.2021.565516)
Supplement: Supplementary file 2 [file Image_2.pdf]

Supplementary Material

1 Supplementary Figures and Tables

1.2. Supplementary Figures

Figure 2. Path Analysis. Model of the influence of the State/Trait Anxiety and Sleep in the executive functioning in children and adolescents during confinement by COVID19.

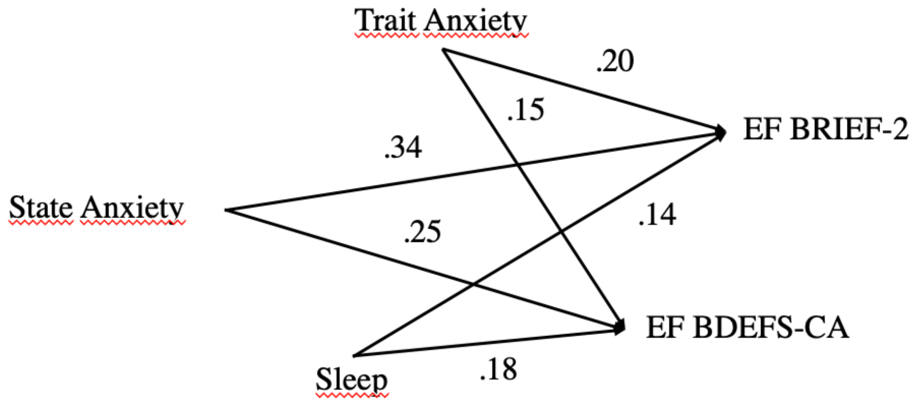

EF BRIEF-2, executive functioning measured with BRIEF-2. Behavioral Evaluation of Executive Function.

EF BDEFS-CA, executive functioning measured with BDEFS-CA. Barkley Deficits in Executive Functioning Scale. Children and Adolescents.
