# Supplementary material for: Consequences of COVID-19 Confinement on Anxiety, Sleep and Executive Functions of Children and Adolescents in Spain
Source: Front Psychol. 2021 Feb 16;12:565516. doi: 10.3389/fpsyg.2021.565516 (PMC7921483; doi:10.3389/fpsyg.2021.565516)
Supplement: Supplementary file 3 [file Table_1.pdf]

Supplementary Material

1 Supplementary Figures and Tables

1.1 Supplementary Tables

Table 1. Means for state/trait anxiety, sleep, and executive functioning according to non-confinement samples.

	**Total Mean (6-18 years) – Non-confinement samples**		**Total Mean – Confinement Sample**	
	State Anxiety	Trait Anxiety	State Anxiety	Trait Anxiety
**Anxiety (STAIC)**	31.2	35.7	34.73	33.67
**Sleep (BEARS)**	7.4		13.18	
**EF (BRIEF-2)**	45.87		42.21	
**EF (BDEFS-CA)**	33.2		70.69	

STAIC. State-Trait Anxiety Inventory for Children.

BEARS. Screening for sleep disorders in childhood.

BRIEF-2. Behavioral Evaluation of Executive Function.

BDEFS-CA. Barkley Deficits in Executive Functioning Scale.Children and Adolescents.
