# Supplementary material for: Consequences of COVID-19 Confinement on Anxiety, Sleep and Executive Functions of Children and Adolescents in Spain
Source: Front Psychol. 2021 Feb 16;12:565516. doi: 10.3389/fpsyg.2021.565516 (PMC7921483; doi:10.3389/fpsyg.2021.565516)
Supplement: Supplementary file 4 [file Table_2.pdf]

Table 2. Student T-test for differences in state anxiety, sleep and executive functions by sex.

Instrument	Sex	N	Mean (SD)	t	p	δ
**STAIC**	Men	548	34.40 (8.47)	-1.32	.19	-
	Women	478	35.12 (9.04)			
**BEARS**	Men	548	13.04 (3.59)	-1.38	.17	-
	Women	478	13.35 (3.67)			
**BRIEF-2**	Men	548	44.70 (16.50)	5.18	.001	.32
	Women	478	39.40 (16.20)			
**BDEFS-CA**	Men	548	76.33 (28.66)	7.05	.001	.44
	Women	478	64.25 (26.21)			

STAIC. State-Trait Anxiety Inventory for Children.
BEARS. Screening for sleep disorders in childhood.
BRIEF-2. Behavioral Evaluation of Executive Function.
BDEFS-CA. Barkley Deficits in Executive Functioning Scale.Children and Adolescents.
