# Supplementary material for: Consequences of COVID-19 Confinement on Anxiety, Sleep and Executive Functions of Children and Adolescents in Spain
Source: Front Psychol. 2021 Feb 16;12:565516. doi: 10.3389/fpsyg.2021.565516 (PMC7921483; doi:10.3389/fpsyg.2021.565516)
Supplement: Supplementary file 5 [file Table_3.pdf]

Supplementary Material

1 Supplementary Figures and Tables

1.1 Supplementary Tables

Table 3. Mann-Whitney U test for differences in executive functions by sex.

**Instrument**	**Sex**	**n**	**Range**	**U**	**p**
**STAIC**	Men	548	512.04	-.17	.87
	Women	478	515.17		

STAIC. State-Trait Anxiety Inventory for Children.
