# Supplementary material for: Consequences of COVID-19 Confinement on Anxiety, Sleep and Executive Functions of Children and Adolescents in Spain
Source: Front Psychol. 2021 Feb 16;12:565516. doi: 10.3389/fpsyg.2021.565516 (PMC7921483; doi:10.3389/fpsyg.2021.565516)
Supplement: Supplementary file 6 [file Table_4.pdf]

Table 4. One way ANOVA for differences in state anxiety, sleep and executive functions according to age group.

Instrument	Age Group	n	Mean (SD)	F	p	ω^2
**STAIC**	6-8	353	33.29 (8.13)			
	9-12	390	35.75 (9.07)	7.41	.001	.01
	13-18	285	35.35 (8.85)			
**BEARS**	6-8	353	12.79 (3.32)			
	9-12	390	13.24 (3.79)	3.83	.02	.01
	13-18	285	13.58 (3.74)			
**BRIEF-2**	6-8	353	42.93 (15.43)			
	9-12	390	42.25 (17.61)	.81	.45	-
	13-18	285	41.25 (16.45)			
**BDEFS-CA**	6-8	353	69.98 (24.37)			
	9-12	390	71.66 (30.50)	.38	.68	-
	13-18	285	70.25 (29.26)			

STAIC. State-Trait Anxiety Inventory for Children.
BEARS. Screening for sleep disorders in childhood.
BRIEF-2. Behavioral Evaluation of Executive Function.
BDEFS-CA. Barkley Deficits in Executive Functioning Scale. Children and Adolescents.
