# Supplementary material for: Consequences of COVID-19 Confinement on Anxiety, Sleep and Executive Functions of Children and Adolescents in Spain
Source: Front Psychol. 2021 Feb 16;12:565516. doi: 10.3389/fpsyg.2021.565516 (PMC7921483; doi:10.3389/fpsyg.2021.565516)
Supplement: Supplementary file 7 [file Table_5.pdf]

Supplementary Material

1 Supplementary Figures and Tables

1.1 Supplementary Tables

Table 5. Kruskal Wallis H test for trait anxiety by age group.

**Instrument**	**Groups**	**n**	**Range**	**H**	**p**
**STAIC**	6-8	353	528.53	2.58	.28
	9-12	390	514.52		
	13-18	285	490.70		

STAIC. State-Trait Anxiety Inventory for Children.
