# Supplementary material for: Consequences of COVID-19 Confinement on Anxiety, Sleep and Executive Functions of Children and Adolescents in Spain
Source: Front Psychol. 2021 Feb 16;12:565516. doi: 10.3389/fpsyg.2021.565516 (PMC7921483; doi:10.3389/fpsyg.2021.565516)
Supplement: Supplementary file 8 [file Table_6.pdf]

Table 6. Differences between males and females with low, average and high state anxiety (SA) in relation to executive functions (BRIEF-2).

**Groups**	**n**	**Mean**	**F**	**Df**	**p**	ω^2
**Men with low SA**	180	34.66	75.52	5	.001	.27
**Men with average SA**	187	44.09				
**Men with high SA**	181	55.33				
**Women with low SA**	149	29.27				
**Women with average SA**	144	38.40				
**Women with high SA**	185	48.33				

SA, state anxiety.
