# Supplementary material for: Consequences of COVID-19 Confinement on Anxiety, Sleep and Executive Functions of Children and Adolescents in Spain
Source: Front Psychol. 2021 Feb 16;12:565516. doi: 10.3389/fpsyg.2021.565516 (PMC7921483; doi:10.3389/fpsyg.2021.565516)
Supplement: Supplementary file 9 [file Table_7.pdf]

Table 7. Differences between males and females with low, average and high state anxiety (SA) in relation to executive functions (BDEFS-CA).

Groups	n	Mean	F	Df	p	ω^2
**Men with low SA**	180	62.49	47.71	5	.001	.19
**Men with average SA**	187	76.19				
**Men with high SA**	181	90.23				
**Women with low SA**	149	51.13				
**Women with average SA**	144	63.82				
**Women with high SA**	185	75.15				

SA, state anxiety.
