# Supplementary material for: Consequences of COVID-19 Confinement on Anxiety, Sleep and Executive Functions of Children and Adolescents in Spain
Source: Front Psychol. 2021 Feb 16;12:565516. doi: 10.3389/fpsyg.2021.565516 (PMC7921483; doi:10.3389/fpsyg.2021.565516)
Supplement: Supplementary file 10 [file Table_8.pdf]

Supplementary Material

1 Supplementary Figures and Tables

1.1 Supplementary Tables

Table 8. Correlations between STAI Cs/Ct scores and the rest of the batteries.

	**BEARS**	**BRIEF-2**	**BDEFS-CA**
**STAICS**	.420	.534	.421
**STAICT**	.024	.384	.387

STAIC. State-Trait Anxiety Inventory for Children.

STAICS, State Anxiety Inventory for Children.

STAICT, Trait Anxiety Inventory for Children.

BEARS. Screening for sleep disorders in childhood.

BRIEF-2. Behavioral Evaluation of Executive Function.

BDEFS-CA. Barkley Deficits in Executive Functioning Scale. Children and Adolescents.
